# Weighted metrics are required when evaluating the performance of prediction models in nested case–control studies

**DOI:** 10.1186/s12874-024-02213-6

**Published:** 2024-05-17

**Authors:** Barbara Rentroia-Pacheco, Domenico Bellomo, Inge M. M. Lakeman, Marlies Wakkee, Loes M. Hollestein, David van Klaveren

**Affiliations:** 1 Department of Dermatology, Erasmus Medical Center Cancer Institute, Erasmus University Medical Center, Dr. Molewaterplein 40, Rotterdam, 3015 GD The Netherlands; 2 SkylineDx B.V, Rotterdam, The Netherlands; 3 Department of Human Genetics, Leiden University Medical Center, Leiden, The Netherlands; 4 Department of Clinical Genetics, Leiden University Medical Center, Leiden, The Netherlands; 5 Department of Research, Netherlands Comprehensive Cancer Organization (IKNL), Utrecht, The Netherlands; 6 Department of Public Health, Center for Medical Decision Making, Erasmus University Medical Center, Rotterdam, The Netherlands

**Keywords:** Prediction model validation, Nested case–control study, Rare outcomes, Weighted metrics

## Abstract

**Background:**

Nested case–control (NCC) designs are efficient for developing and validating prediction models that use expensive or difficult-to-obtain predictors, especially when the outcome is rare. Previous research has focused on how to develop prediction models in this sampling design, but little attention has been given to model validation in this context. We therefore aimed to systematically characterize the key elements for the correct evaluation of the performance of prediction models in NCC data.

**Methods:**

We proposed how to correctly evaluate prediction models in NCC data, by adjusting performance metrics with sampling weights to account for the NCC sampling. We included in this study the C-index, threshold-based metrics, Observed-to-expected events ratio (O/E ratio), calibration slope, and decision curve analysis. We illustrated the proposed metrics with a validation of the Breast and Ovarian Analysis of Disease Incidence and Carrier Estimation Algorithm (BOADICEA version 5) in data from the population-based Rotterdam study. We compared the metrics obtained in the full cohort with those obtained in NCC datasets sampled from the Rotterdam study, with and without a matched design.

**Results:**

Performance metrics without weight adjustment were biased: the unweighted C-index in NCC datasets was 0.61 (0.58–0.63) for the unmatched design, while the C-index in the full cohort and the weighted C-index in the NCC datasets were similar: 0.65 (0.62–0.69) and 0.65 (0.61–0.69), respectively. The unweighted O/E ratio was 18.38 (17.67–19.06) in the NCC datasets, while it was 1.69 (1.42–1.93) in the full cohort and its weighted version in the NCC datasets was 1.68 (1.53–1.84). Similarly, weighted adjustments of threshold-based metrics and net benefit for decision curves were unbiased estimates of the corresponding metrics in the full cohort, while the corresponding unweighted metrics were biased. In the matched design, the bias of the unweighted metrics was larger, but it could also be compensated by the weight adjustment.

**Conclusions:**

Nested case–control studies are an efficient solution for evaluating the performance of prediction models that use expensive or difficult-to-obtain biomarkers, especially when the outcome is rare, but the performance metrics need to be adjusted to the sampling procedure.

**Supplementary Information:**

The online version contains supplementary material available at 10.1186/s12874-024-02213-6.

## Background

Risk prediction models are becoming increasingly popular in the medical community to predict clinical outcomes and can be used to provide more personalized decisions to patients. Population-based longitudinal cohorts, with information on thousands of individuals, are now widespread. These cohorts enable the identification of individuals with a disease and their biological samples at the population level, as well as the development of (risk) prediction models [1, 2]. While population-based cohorts are the preferred study design for building and validating such models [3, 4], they are expensive, particularly when the collected data go beyond routinely available variables (e.g. demographic or basic clinical characteristics), and include additional information (e.g. measurements of expensive or difficult-to-obtain biomarkers). In addition, collecting this information for all subjects might not always be feasible. Using the full cohort is, therefore, functional for unbiased sample identification, but not efficient for model development and validation, particularly for rare outcomes. These scenarios require more efficient study designs, such as nested case–control (NCC) and case-cohort designs [3, 4].

In NCC studies, a subsample of a fully enumerated source population is identified, containing all patients who experience the outcome of interest during the study follow-up period (cases), together with a sample of patients who do not experience the outcome during the time-at-risk (controls) [4, 5]. Often, controls are matched to cases to reduce potential confounding [5]. Follow-up and matching variables must be available in the full cohort to identify the controls, whereas other variables of interest are only collected for the case–control set. This greatly reduces the amount of data collection needed (and the associated costs), while still providing accurate estimates of the effects of risk factors and performance estimates [4, 6]. In contrast to the traditional case–control design, where cases and controls are sampled from a population of unknown size, in NCC studies, cases and controls are sampled from a well-defined population of known size (i.e. hence the designation “nested”) [3]. This makes them suitable to estimate hazard and odds ratios of the full cohort and to build absolute risk models [3, 5], without the need for additional data sources. However, appropriate methodology must be employed to accommodate the under-sampling of controls and potentially any matching [5, 7, 8].

Clear recommendations on how to develop prediction models in NCC designs can be found in the literature [9, 10], as well as some examples of risk prediction models for rare outcomes developed in NCC data [8, 11–13]. However, we have noticed that there is a lack of clear guidance on how to properly validate prediction models in this study design. This has led to incorrect performance estimates of these models reported in several works in the literature [14, 15], and to the misconception that NCC studies are not suited to validate prediction models [16]. Clear guidance on this matter would therefore be useful for clinical and methodological researchers evaluating the performance of prediction models in NCC data.

We aim to present the key elements for correct evaluation of the performance of prediction models in NCC datasets. We propose how relevant model performance metrics should be adjusted to compensate for 1) the overrepresentation of cases in the NCC dataset and 2) the fact that the controls in the NCC dataset are no longer representative of all controls in the source population. We then illustrate the importance of using adjusted performance metrics with a real world example, where we validate the well-known BOADICEA [17] breast cancer risk prediction model in NCC datasets, and compare the obtained performance metrics to those that were reported in the original full cohort study [18].

## Methods

### Validating risk prediction models in nested case–control data

**Fig. 1:**
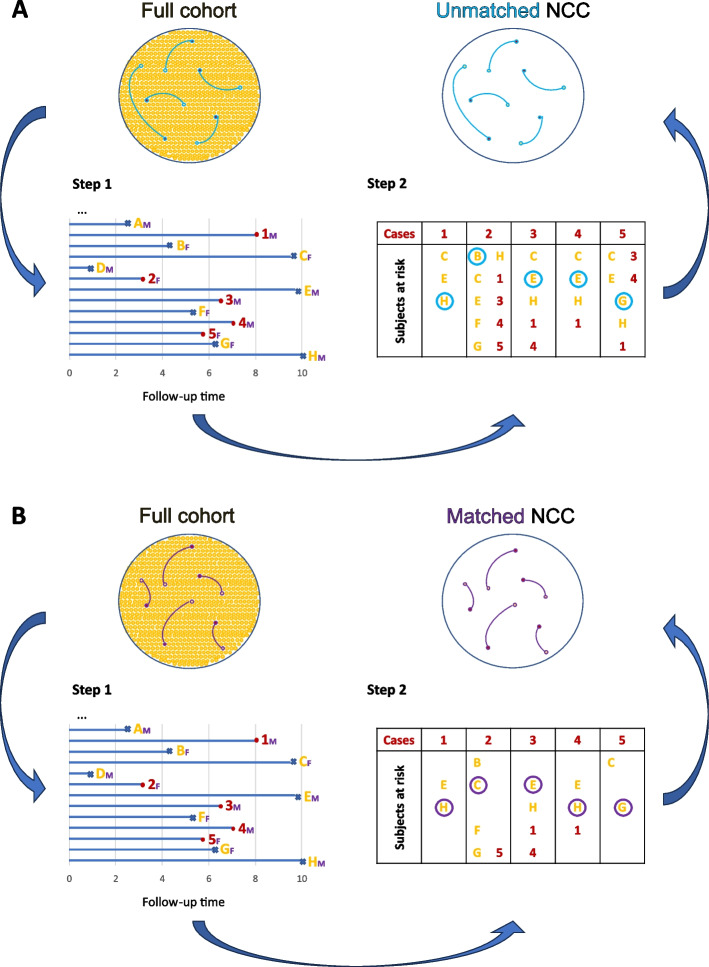
Derivation of a nested case–control dataset from a full cohort, using incidence density sampling. In **A**, no matching variable is used. In **B**, sex is used to match controls to cases. Step 1. Required information for all subjects in the source population (full cohort) is extracted: outcome of interest, follow up time and, if needed, additional matching variables. Time-to-event plot represents timelines of subjects in the full cohort (each row represents one subject). Cases (in red) correspond to subjects who experience the outcome of interest. Controls (in yellow) correspond to subjects who do not experience the outcome of interest. Crosses represent last time of follow-up for controls. Sex (male (M) or female (F)) is indicated in purple. Step 2: Control sampling using incidence density sampling: for each case, the subjects who have not experienced any event at the time of the event for that case (subjects-at-risk) are identified, and one or more subjects-at-risk are randomly sampled. In this example, the sampling ratio is 1:1, therefore only 1 control is sampled per case (indicated with a light blue circle in **A** and a purple circle in **B**). The number of subjects-at-risk in step 2 in the matched scenario (**B**) is typically smaller than in the scenario without matching (**A**). Note that, under incidence density sampling, some cases can be sampled as controls if they are part of the subjects-at-risk for another case before they experience the event of interest. Subjects can also be sampled more than once as controls. Other sampling methods exist, but incidence density sampling is the method that leads to more unbiased estimates in NCC datasets

In this study, we focus on the scenario where a prediction model has already been developed, in a population-based cohort or in another design, and we aim to evaluate its performance in an NCC design. Table 1 describes how NCC datasets are obtained using incidence density sampling, and Fig. 1 illustrates two completely worked out examples for an unmatched (A) and matched design (B) respectively. Of note, under this sampling method, the same subject can be selected multiple times: cases can be selected as controls of other cases with shorter event times; controls can be selected in risk sets of different cases. However, for model validation in NCC datasets, including the same subject multiple times can lead to biased estimates of performance metrics [4]. Therefore, only one record should be kept for each subject: the case record for subjects selected as cases, and a randomly selected control record for controls [4].

**Table 1:** Sampling of a nested case–control (NCC) dataset from a full cohort

In NCC studies, a subsample of a fully enumerated source population (full cohort) is identified. This subsample contains all patients who have experienced the outcome of interest during the study follow-up period (*cases*), together with a sample of patients who have not experienced the outcome during the time-at-risk (*controls*). Often, controls are matched to cases on some additional variables such as sex or age. Steps to obtain an unmatched or matched NCC dataset are similar (Fig. 1): first, required information for all subjects in the source population is extracted: outcome of interest, follow up time and, if needed, additional matching variables. Second, a pre-defined number of controls is sampled for each case, preferably using the incidence density sampling method [5]. This sampling step is more constrained when matching is applied

### Challenges in validating prediction models in NCC data

**Fig. 2:**
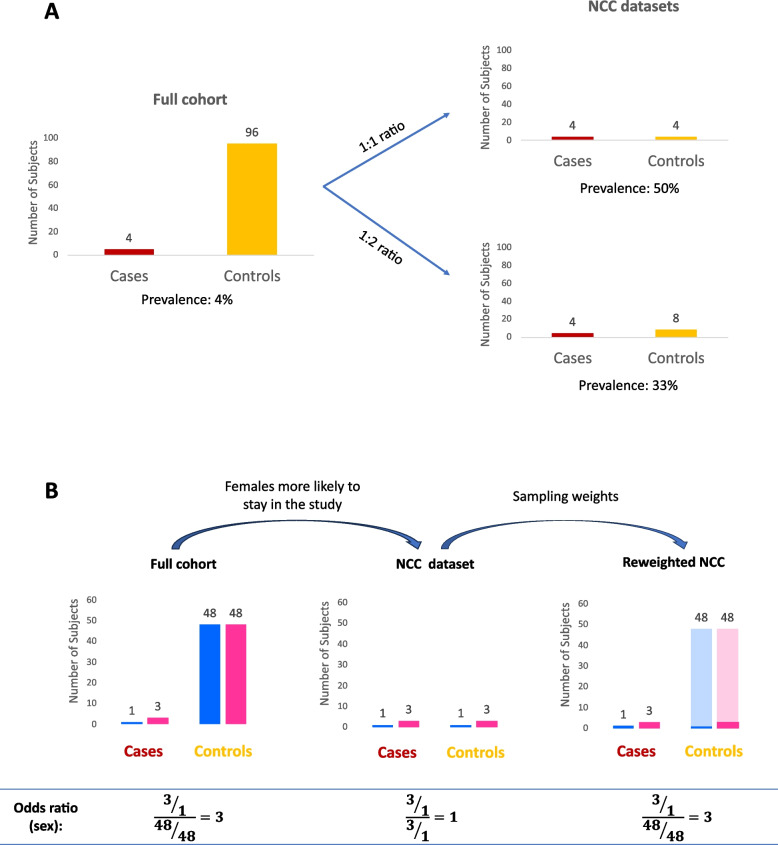
Characteristics of nested case–control (NCC) datasets that affect model validation (**A**) Comparison of proportions of cases (in red) and of controls (in yellow) in the full cohort and the NCC data. Outcome prevalence is distorted in the NCC dataset, compared to the full cohort. The magnitude of the distortion depends on the sampling ratio (number of controls sampled for each case). (**B**) NCC sampling can distort risk factor distribution in sampled controls. For example, we might be studying a clinical outcome that is more prevalent in females (in pink) than males (in blue), with 75% of events occurring in females (3 out of 4 events). However, in subjects without events sex is evenly distributed (48 females + 48 males). In the study, males are more likely to leave the study earlier than females, leading to shorter follow up times for males. This results in females being more likely to be sampled as controls: the proportion of females in the NCC controls is 75% instead of 50% observed in the full cohort. Reweighting the NCC data can reconstruct the original distribution of sex in the sampled controls (50% males, 50% females) and allow the unbiased estimation of odds ratios and performance metrics

Validating prediction models in NCC datasets cannot be performed in the same way as in full cohorts for two reasons. First, the sampling procedure artificially increases the proportion of cases (Fig. 2A). The difference between the original proportion and the one in the NCC dataset will depend on the chosen sampling ratio (number of controls sampled per case). Second, the controls in the NCC dataset are no longer representative of all controls: even if no matching is performed, subjects with longer survival time are more likely to be included in the NCC dataset as controls. This can distort the distributions of the risk factors in the NCC dataset (Fig. 2B). Failing to account for this distortion will result in overestimation of the absolute risk, since controls are underrepresented in the NCC dataset compared to the full cohort, and in the inaccurate estimation of both risk factor effects (e.g., odds ratios) and performance metrics.

However, if subjects are weighted based on their sampling probability, NCC datasets can be used to develop and validate prediction models. The weighting process enables the recovery of the original ratio of cases to controls and the original risk factor distributions, which consequently enables the development of models that predict absolute risk [5, 10]. Additionally, in the validation of a model in an NCC dataset, the same weighting process can be applied to adjust the performance metrics, making it possible to evaluate model performance accurately, without applying the model to all subjects of the corresponding full cohort.

### Assigning weights to subjects in NCC data

The weight of subject $k$ ${(w}_{k})$ is computed as the inverse of the probability of subject $k$ being included in the NCC dataset, and accounts for the sampling design, namely any matching performed, as well as the case–control sampling ratio. For a NCC dataset with $K$ subjects ($J$ cases and $I$ controls), the sampling probability of case $j$ ${(p}_{j})$ is straightforward to compute. However, the sampling probabilities of controls, each denoted as ${p}_{i}$, take a more complex form, as several factors can influence their selection.

#### Sampling probabilities of cases (${{\varvec{p}}}_{{\varvec{j}}}$)

In a typical NCC study, all cases present in the full cohort are included in the NCC dataset; therefore, all cases have a sampling probability ${p}_{j}$ of 1. However, extracting model variables for all cases might not be possible due to time or cost constraints, leading to more atypical NCC studies, where only a portion of the cases are included. In this scenario, the sampling probability corresponds to the proportion of cases identified in the full cohort that are included in the NCC dataset [19].

#### Sampling probabilities of controls (${{\varvec{p}}}_{{\varvec{i}}}$)

There are different ways of estimating the probability of sampling subject $k$ as a control [20]. We focus on the Kaplan–Meier and the logistic/generalized additive model estimators, for their ease of implementation and ability to deal with matched NCC designs.

The sampling probability of control $i$ (${p}_{i}$) estimated with the Kaplan–Meier estimate [20] uses the property that under the incidence density sampling scheme, the probabilities of a control being eligible for sampling for different cases are independent across the (matched) risk sets [21]. It is defined as follows:

1 $$\begin{aligned}{p}_{i}& = 1-p\left(i \text{ is not sampled as control for any case}\right)\quad \\&= 1-\prod_j^ \space p\left(i \text{ is not sampled as control for any case }j\right)\\ \quad \\&= 1-\prod_j^ \space (1-\frac{m}{n_{j}(t_{j})-1}I (\text{Control } i \text{ is eligible for sampling for case }j))\end{aligned}$$

where $m$ is the number of controls sampled per case, $I$ is an indicator variable (with value 1 if control $i$ is eligible for case $j$, and 0 otherwise), ${t}_{j}$ is the event time of case $j$, and ${n}_{j}\left({t}_{j}\right)$ is the number of subjects at risk at time ${t}_{j}$, which could be matched to case $j$.

Sampling probabilities can also be estimated using a model-based approach. For example, a logistic regression model can be fitted to predict the probability of a control being sampled, using its censoring time and additional matching variables as predictors. This logistic regression is fitted to the full cohort, excluding all cases. The sampling probability ${p}_{i}$ then corresponds to the output probability of this logistic regression applied to control $i$, and the inverse of such sampling probabilities are denominated generalized linear model (GLM) weights. Sampling probabilities can also be estimated based on a generalized additive model (GAM), leading to GAM weights.

These weighting schemes are described in more detail in Støer and Samuelsen [20]. Of note, other weighing methods exist, such as the Chen weights [20]. Despite their differences, there is little variation in model estimates and standard errors using different weighting schemes during model development [10, 22]. All of these methods for weight computation can be implemented with the *multipleNCC* R package [20].

#### Inverse probability weights

Once the sampling probabilities are computed, the weight ${w}_{k}$ assigned to subject $k$ is:

2 $${w}_{k}=\frac{1}{{p}_{k}}$$

In both typical and atypical NCC data, the weights of all cases have the same value, because the sampling probability is the same for all cases. The weights of controls depend on the control and are always larger (or equal) to 1, in order to represent multiple controls in the original cohort: the lower their probability of being sampled, the higher their weight [20].

Of note, the sum of the weights will generally not correspond to the number of subjects in the cohort, particularly if KM-type of weights are used. To compensate for this, the weights of controls should be rescaled [4]:

3 $${w}_{k}{\prime}= {w}_{k}\times \frac{{n}_{c}}{\sum_{i=1}^{{n}_{c}}{w}_{i}}$$

where ${w}_{k}{\prime}$ is the rescaled version of weight ${w}_{k}$ and ${n}_{c}$ is the total number of controls in the cohort.

### Model performance metrics adjusted to the NCC data

As recommended in the TRIPOD guidelines for risk prediction models [23], models should be validated with respect to their discrimination ability, the agreement between observed and predicted outcomes (calibration), and the clinical utility they provide. Therefore, we focused on the following performance metrics to quantify these characteristics: C-index, threshold-based metrics (such as sensitivity and specificity), observed-to-events ratio, calibration slope, and decision curve analysis. The application of these metrics to full cohorts is straightforward, and we refer the reader to the literature for an in depth characterization of these metrics [24, 25]. While these metrics can be obtained in NCC studies, they must be adjusted due to the under-sampling of controls, by using the weights of the subjects included in the NCC dataset. Of note, adjusted metrics should be used in both internal or external validation of the models in NCC datasets (see the section “Bootstrapping and cross-validation in NCC data”).

#### C-index

The C-index estimates the probability that the predicted order of the events of a randomly selected subject pair is correct. The C-index is calculated by identifying all possible pairs where at least one subject had an event (usable pairs). From these, concordant pairs and discordant pairs are identified as pairs where subjects with longer survival time have smaller or larger predicted risk, respectively. The C-index is then calculated as follows:

4 $$C-index=\frac{\sum_{k}{C}_{k}}{\sum_{k}{(D}_{k}+{C}_{k})}$$

where ${C}_{k}$ and ${D}_{k}$ denote respectively the number of concordant and discordant pairs for subject $k$.

When a model is validated in an NCC dataset, the C-index should be adjusted by weighing the concordant and discordant pairs by the estimated weight of subject $k$ (${w}_{k}$), as follows [4]:

5 $${\mathit{weighted\ C}}-index=\frac{\sum_{k}{{w}_{k}C}_{k}}{\sum_{k}{w}_{k}{(D}_{k}+{C}_{k})}$$

#### Threshold-based performance metrics

Often, prediction models are used with decision thresholds: subjects above and below the risk threshold are considered high-risk and low-risk, respectively. Performance metrics such as sensitivity (SE), specificity (SP), positive predictive value (PPV) and negative predictive value (NPV) are informative to validate a model in this setting. The need to adjust PPV and NPV is clear, as these metrics are directly dependent on the prevalence of the outcome of interest in a cohort [26], but SE and SP must also be adjusted in NCC datasets, due to the biased sampling. Unbiased estimates of these metrics are obtained by using the weights of cases and controls as described in Table 2.

**Table 2:** Definition of performance metrics to validate models with decision thresholds, and respective modifications for NCC data

Threshold-based metrics on NCC data	
**Binary data**	**Survival data**
$SE=\frac{TP}{Pos}$	$SE=\frac{TP}{Pos}$
$SP=\frac{TN}{Neg}$	$SP=\frac{TN}{Neg}$
$PPV=\frac{TP}{TP+FP}$	$PPV=1-\left({S}_{w}\left(t\right)|{\text{r}}>{p}_{t}\right)$
$NPV=\frac{TN}{TN+FN}$	$NPV=\left({S}_{w}\left(t\right)|r\le {p}_{t}\right)$
Where:	
$TN=\sum_{k=1}^{n}{w}_{k}I\left({x}_{k}=0,{r}_{k}\le {p}_{t}\right)$	$TN=\left({S}_{w}\left(t\right)|r\le {p}_{t}\right)\times \sum_{k=1}^{n}{w}_{k}I({r}_{k}\le {p}_{t})$
$FP=\sum_{k=1}^{n}{w}_{k}I\left({x}_{k}=0,{r}_{k}>{p}_{t}\right)$	$FP= \left({S}_{w}\left(t\right)|{\text{r}}>{p}_{t}\right)\times \sum_{k=1}^{n}{w}_{k}I({r}_{k}>{p}_{t})$
$TP=\sum_{k=1}^{n}{w}_{k}I\left({x}_{k}=1,{r}_{k}>{p}_{t}\right)$	$TP=\left[1-\left({S}_{w}\left(t\right)|{\text{r}}>{p}_{t}\right)\right]\times \sum_{k=1}^{n}{w}_{k}I({r}_{k}>{p}_{t})$
$FN=\sum_{k=1}^{n}{w}_{k}I\left({x}_{k}=1,{r}_{k}\le {p}_{t}\right)$	$FN=[1- \left({S}_{w}\left(t\right)|r\le {p}_{t}\right)]\times \sum_{k=1}^{n}{w}_{k}I({r}_{k}\le {p}_{t})$
$Pos= TP+FN$	$Pos= [1- {S}_{w}\left(t\right)]\times \sum_{k=1}^{n}{w}_{k}$
$Neg= TN+FP$	$Neg= [ {S}_{w}\left(t\right)]\times \sum_{k=1}^{n}{w}_{k}$

$SE$ Sensitivity,$SP$ Specificity,$PPV$ Positive predictive value,$NPV$ Negative predictive value,$TP$ number of true positives,$TN$ number of true negatives,$FP$ number of false positives,$FN$ number of false negatives,${p}_{t}$ threshold probability,$r$ risk probability, *n *total number of subjects in the NCC dataset

**Indicator variables:**

$I\left({x}_{k}=1\right)$ is an indicator variable = 1 if subject $k$ is a case, and 0 otherwise

$I\left({x}_{k}=0\right)$ is an indicator variable = 1 if subject $k$ is a control, and 0 otherwise

$I\left({r}_{k}\le {p}_{t}\right)$ is an indicator variable = 1 if subject $k$ has a predicted risk probability $\le {p}_{t}$, and 0 otherwise

$I\left({r}_{k}>{p}_{t}\right)$ is an indicator variable = 1 if subject $k$ has a predicted risk probability > ${p}_{t}$, and 0 otherwise

**Survival data:**

$\left({S}_{w}\left(t\right)|{\text{r}}>{p}_{t}\right)$ is the weighted Kaplan–Meier survival probability for subjects with a predicted risk probability $>{p}_{t}$. Weighted Kaplan–Meier estimates are obtained by using the sum of weights of individuals with an event at time t, instead of the number of events at that time; and the sum of the weights of the subjects-at-risk at time t, instead of the number of subjects-at-risk at that time

$\left({S}_{w}\left(t\right)|r\le {p}_{t}\right)$ is the weighted Kaplan–Meier survival probability for subjects with a predicted risk probability $\le {p}_{t}$

Competing risks can be incorporated by replacing ${S}_{w}\left(t\right)$ with the complement of a weighted cumulative incidence function

Note: The definitions of $Pos$, $Neg$, $PPV$ and $NPV$ are provided for binary and survival data separately, to avoid computing Kaplan–Meier survival estimates for the quantities $TN,FP,TP$ and $FN$ and then adding these up, which produces slightly different results than directly estimating survival probabilities of the larger groups, such as $TP+FN$

The adjustment of these metrics has been described for model validation in NCC datasets when the outcome of interest is binary, and no survival data was available [6]. In this situation, the weights of all controls are the same and they correspond to the inverse of the control sampling fraction (i.e., $\frac{1}{\frac{sampled \space controls}{total \space number \space of \space controls}}$). In Table 2, we generalized this adjustment for survival data and for the possibility of controls having different weights.

#### Calibration

Model calibration aims to assess whether the absolute risks predicted by the model correctly estimate the observed risks. For example, for patients with, say, a 20% predicted risk of an event of interest, 20 patients out of 100 should indeed experience the event.

Calibration can be evaluated in multiple ways. We focused on the most important calibration metrics for risk prediction models [27]: mean calibration, calibration slope, and calibration plot.

Mean calibration corresponds to the difference between the proportion of observed events, for binary events; or 1-observed survival fraction at the chosen time point estimated by e.g. the Kaplan–Meier estimate, for survival data [28], and the average predicted risk. It can also be expressed as a ratio of observed to expected (i.e., predicted) events. In a NCC dataset, the observed events or survival fraction should be derived from the full cohort, and, apart from rounding differences, these should correspond to the weighted event proportion or weighted KM survival probability, respectively. Likewise, the average predicted risk should be computed using the weighted average of the predicted risk, with the previously described weights.

The calibration slope summarizes the strength of the association between predicted and observed outcomes [27]. It corresponds to the slope of the regression of the observed outcomes (binary or survival) on the linear predictor of the model. A perfectly calibrated model has a slope of 1, while lower and greater values indicate over- and underfitting respectively. Calibration can be visualized in a calibration plot showing the observed proportions or (1-observed survival fractions) against the average predicted risks for a given number of subject groups. For NCC datasets, a weighted regression of the observed outcomes (binary or survival) on the linear predictors of the model should be used to obtain the calibration slope (Supplementary Table 1). The calibration plot should show weighted observed proportions/survival fractions against weighted averages of the predicted risks for a given number of subject groups.

#### Decision curve analysis

Discrimination and calibration measures are insufficient to evaluate the model utility in the context of a clinical decision. Decision curve analysis aims to assess the net benefit of making clinical decisions based on the prediction model compared to other models or default strategies by accounting for the trade-off between the relative harms of false positives and false negatives [29]. The relative harm can be interpreted as how much worse it is to miss a poor clinical outcome and wrongly withholding treatment (i.e., false negative) compared to providing an unnecessary intervention to a patient who will not develop the outcome (i.e., false positive). For example, if the harm of missing a cancer is 4 times greater than performing an unnecessary invasive procedure, we can translate this relative harm into a probability threshold such that the patient should only undergo the procedure if the risk is greater than the threshold (1/(4 + 1) = 0.2). The decision curve is constructed by computing the net benefit over a clinically meaningful range of probability thresholds.

The net benefit (NB) at a given threshold is computed as follows [29]:

6 $$NB(t)=\frac{TP}{n}-\frac{FP}{n}(\frac{{p}_{t}}{1-{p}_{t}})$$

where $TP$ denotes true positives, $FP$ denotes false positives, $n$ is the total number of subjects and ${p}_{t}$ is the probability threshold.

In NCC data, the net benefit of Eq. (6) should be calculated using the definitions of $TP$ and $FP$ for binary or survival data provided in Table 2. Net benefit for binary outcomes in NCC data has already been derived in [30], but not for survival outcomes.

### Bootstrapping and cross-validation in NCC data

Bootstrapping and cross-validation are techniques that are frequently employed to internally validate prediction models and to obtain estimates of the variability of model performance metrics. In general, bootstrap samples and cross-validation folds should mimic the original sampling design as well as possible [31]. Therefore, when these procedures are applied to NCC datasets, they should account for the fact that the NCC design is a stratified design, where controls are matched to cases (at least on follow-up time). This translates into obtaining bootstrap samples by sampling case–control pairs with replacement, rather than individual samples. In the case of cross-validation, case–control pairs should be included in the same fold in cross-validation schemes. Weighted metrics should also be used when evaluating the model within the NCC cross-validation folds and the bootstrap samples.

#### Code availability

We implemented a subset of the weighted performance metrics (threshold-based metrics, calibration plot and decision curve analysis for survival data). We used the R packages described in Supplementary Table 1 for computing sampling weights and the remaining weighted performance metrics. The code to reproduce our analysis is available at https://github.com/emc-dermatology/ncc-evaluation.

## Real-world illustration: validation of the BOADICEA model

Breast cancer is the most commonly diagnosed cancer in women worldwide [32]. Identifying high-risk individuals is critical to reduce mortality and improve quality of life. However, excessive screening also results in false positives and overdiagnosis [33]. Risk prediction models can help healthcare systems by targeting women at high risk of developing breast cancer for screening, while reducing the side-effects of screening for those at lower risk.

In this clinical illustration, we focused on the validation of the Breast and Ovarian Analysis of Disease Incidence and Carrier Estimation Algorithm (BOADICEA version 5) model in the Rotterdam study, a population-based cohort. The performance of the model predicting the development of breast cancer (invasive or in situ) within 10 years has been previously evaluated in this cohort [18]. We use this cohort to show that it is also possible to validate a prediction model in a sub-cohort much smaller than the full cohort (more than 10 times smaller in this case), if the sub-cohort is carefully designed (i.e., the nested case–control dataset).

### Model and study cohort

The BOADICEA model is an absolute risk prediction model that estimates the probability of developing breast cancer within 5 years, 10 years or within a lifetime, for women until the age of 80. The model uses genetic input variables such as a polygenic risk score (PRS) based on 313 breast cancer associated variants, together with nongenetic risk factors and family history. The nongenetic risk factors are: mammographic density, age at menarche, age at menopause, parity, age at first live birth, oral contraceptive use, hormonal treatment, height body mass index and alcohol intake. The formula for computing the absolute risk is available in Lee et al. [17]. The model was validated in the Rotterdam Study cohort, a population-based cohort of elderly Dutch individuals living in a district of Rotterdam in the Netherlands. In 2008, the cohort consisted of 14,926 subjects aged 45 years or older, out of which 8,823 were women. As in Lakeman et al. [18], we excluded all women for whom 10-year BOADICEA risk estimates could not be obtained: women for whom genotype data were not available or did not have enough quality (*n* = 2153), and women who, at the time of recruitment into the cohort, had breast cancer (*n* = 148) or were older than 70 years (*n* = 2145). This left a total of 4,377 women. All BOADICEA risk factors were available in this cohort, except for mammographic density. However, the BOADICEA model allows for missing information [17].

### Ethics statement

The Rotterdam Study has been approved by the institutional review board (Medical Ethics Committee) of the Erasmus Medical Center and by the review board of The Netherlands Ministry of Health, Welfare and Sports. The analyses in this study were approved by the management team of the Rotterdam study.

### Illustration setup

NCC datasets with one control per case were derived from the full cohort of 4,377 subjects in 3 different scenarios: a NCC design without any matching variables (NCC-NM); an NCC design with matching on an administrative variable (Rotterdam study sub-cohort), which is not strongly associated with the model predictions (NCC-MNR); and an NCC design with matching based on the non-genetic risk estimates (NCC-MR). In the NCC-MNR design, the matching variable has 3 categories, and in the NCC-MR, the matching variable has 6 categories, representing the risk percentages (from 1 to 6%). Control sampling in all designs was performed with incidence density sampling without duplicated subjects. Cases without a control after duplicate removal were matched to another control so that the total KM weight sum before rescaling was similar to the size of the full cohort. Control replacement is typically possible for NCC designs in rare outcomes and sufficiently loose matching.

The BOADICEA model was applied to both the full cohort and all the derived NCC datasets (Supplementary Fig. 1). Performance metrics, including the C-index, calibration metrics, and decision curve analysis, were computed in all cohorts. For the NCC datasets, the performance of the BOADICEA model was evaluated with and without adjustment for the NCC design. These unweighted (“naïve”) and weighted metrics were compared with the performance metrics on the full cohort. In practice, only one NCC dataset would be sampled, and performance metrics would be evaluated on that single cohort, however, to account for sampling variation, we repeated the derivation of NCC datasets 100 times in each scenario. As a sensitivity analysis, we used three ways to compute the sampling weights: Kaplan–Meier type of weights (KM), weights computed with logistic regression (GLM) and weights computed with generalized additive models (GAM). We also repeated the analyses with NCC datasets with 2 controls per case, to investigate the impact of the case–control ratio on the precision of the performance metric estimates.

## Results

A total of 4,377 subjects were included in our analysis, out of which 163 developed breast cancer within 10 years of their recruitment into the cohort (Table 3). The median follow-up was 12.9 (6.9–20.9) years for subjects without an event. Women who developed breast cancer (cases) differed significantly from those who did not (controls) in Body Mass Index and in their 313-variant PRS. The original Rotterdam study (RS-I) was extended twice (RS-II and RS-III) [34]. The proportion of cases was significantly different among these sub-cohorts.

**Table 3:** Characteristics of the subjects in the Rotterdam study included in our real-world illustration. Only subjects for whom the 10-year BOADICEA risk predictions could be obtained (women younger than 70 years, with genotype data of sufficient quality) were included. This cohort is designated as the “full cohort” in our illustration. A more extensive description of the patient characteristics is provided in Lakeman et al. [18] Mammographic density was lacking for all subjects

Variables		Controls, *N* = 4214^a^	Cases, *N* = 163^a^	*p*-value^b^
Administrative	Study recruitment			< 0.001
	1st (RS-I)	1881 (45%)	92 (57%)	
	2nd (RS-II)	808 (19%)	38 (23%)	
	3rd (RS-III)	1525 (36%)	33 (20%)	
	Follow-up, in years	12.9 (6.9, 20.9)	5.1 (2.3, 7.2)	< 0.001
	Year of birth	1938 (1930, 1948)	1935 (1929, 1942)	0.003
	Age at recruitment	60.0 (56.7, 63.4)	60.9 (57.2, 64.5)	0.15
Risk factors	Height, in m missing (%)	1.64 (1.60, 1.68) 9 (0.2%)	1.64 (1.61, 1.68) 3 (1.8%)	0.43
	Age at menarche missing (%)	13 (12, 14) 102 (2.4%)	13 (12, 14) 4 (2.5%)	0.52
	Age at menopause missing (%)	50 (46, 53) 255 (6.1%)	51 (47, 53) 15 (9.2%)	0.12
	Number of children missing (%)	2 (1, 3) 584 (13%)	2 (1, 3) 33 (20%)	0.74
	Age at first childbirth^c^ missing (%)	24 (22, 27) 19 (0.5%)	25 (23, 28) 1 (0.6%)	0.06
	Any hormonal treatment missing (%)	758 (18%) 40 (1%)	32 (20%) 3 (1.8%)	0.55
	Any oral contraception missing (%)	2665 (68%) 311 (7.4%)	90 (64%) 23 (14%)	0.32
	Body Mass Index missing (%)	26.3 (23.9, 29.4) 38 (0.9%)	27.5 (24.7, 30.5) 3 (1.8%)	0.01
	Alcohol use, in grams per day missing (%)	3 (0, 9) 742 (18%)	3 (0, 9) 34 (21%)	0.98
Genetic	Standardized PRS	0.05 (-0.61, 0.72)	0.62 (-0.04, 1.35)	< 0.001
10y risk estimates (%), based on	Age	2.9 (2.7, 3.3)	2.9 (2.7, 3.1)	0.02
	Age + RF	2.4 (2.0, 2.9)	2.5 (2.2, 3.0)	0.11
	Age + PRS	2.9 (2.2, 3.7)	3.5 (2.7, 4.4)	< 0.001
	Age + RF + PRS	2.3 (1.7, 3.2)	3.0 (2.3, 4.2)	< 0.001

*RS-I* Original Rotterdam study cohort

*RS-II, RS-III* Extensions of the Rotterdam study cohort

*RF* Non-genetic risk factors

*PRS* Polygenic risk score

^a^Median (Interquantile range); *n* (%)

^b^Wilcoxon rank sum test for numerical variables; Pearson's Chi-squared test for categorical variables

^c^For women known to have children

### Model validation in the full cohort

**Fig. 3:**
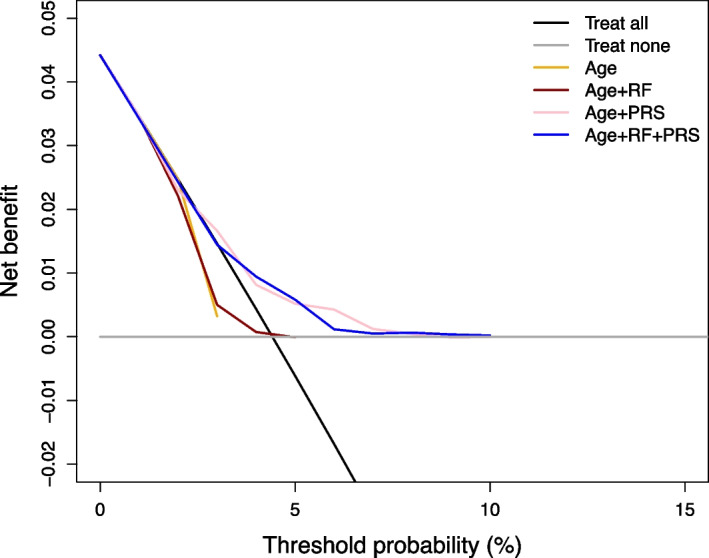
Decision curve analysis comparing different components of the BOADICEA model in the full cohort of 4377 women. The plot describes the decision curves for 1) the 10-year risk estimates based on Age alone (Age); 2) Age and Risk Factors (Age + RF); 3) Age and Polygenic Risk Score (Age + PRS); and, finally, for 4) the full BOADICEA model, with all these components (Age + RF + PRS). Risk factors do not include mammography density information

Similarly to the validation publication in the Rotterdam Study [18], the performance of the BOADICEA model was evaluated in all the subjects of this cohort, and compared to the performance of the risk estimates based on subsets of the model components (age only, age and non-genetic risk factors, and age and genetic component). All of the metrics are reported in Supplementary Table 2). The BOADICEA model (using age, non-genetic risk factors and the PRS) showed reasonable discriminative ability (C-index 0.65 (0.61–0.69)) and calibration (calibration slope 1.19 (0.87–1.54)). However, breast cancer occurrences were substantially underestimated, with an observed-to-expected ratio (O/E ratio) of 1.69 (1.42–1.93). This underestimation was more evident in the higher risk range (Supplementary Fig. 2). Despite the observed miscalibration, decision curve analysis showed that the BOADICEA model is still clinically useful for targeted screening in the vicinity of the outcome cumulative incidence (Fig. 3): for threshold probabilities between 3% and 7.5%, the model outperformed the treat all strategy. Interestingly, comparison of different subcomponents of the model shows that risk estimates based on the genetic component were more clinically useful than risk estimates relying on age or risk factors alone. Since risk predictions are often dichotomized to guide decisions, we used the risk threshold described in the BOADICEA development publication [17] (3%) and classified subjects with a risk prediction lower than 3% as low-risk and the remaining as high-risk. Performance metrics obtained with this risk threshold were suboptimal: namely, sensitivity to detect breast cancer occurrences was only 0.52 (0.43–0.61).

### Model validation in NCC data

**Fig. 4:**
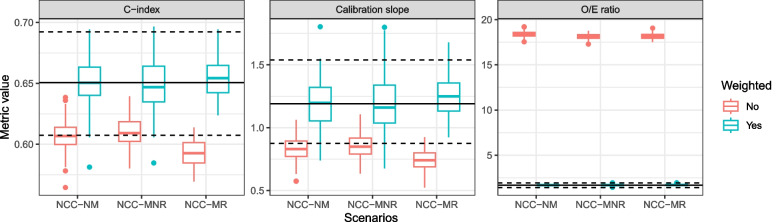
Performance metrics obtained in the full cohort and in the NCC datasets, for threshold-independent model predictions. The horizontal black line indicates the value of the performance metric in the full cohort and the dashed horizontal black lines indicate the upper and lower bounds of the 95% confidence interval of the performance metric in the full cohort. The color of the boxplots indicates whether performance metrics on the NCC datasets are weighted (“Yes”) or unweighted (“No”). NCC-NM: a regular NCC design with incidence density sampling and without any matching variables; NCC-MNR: a NCC design with incidence density sampling and matching on an administrative variable, which is not associated with the model predictions; NCC-MR: NCC design with incidence density sampling and matching based on the non-genetic risk predictions

**Fig. 5:**
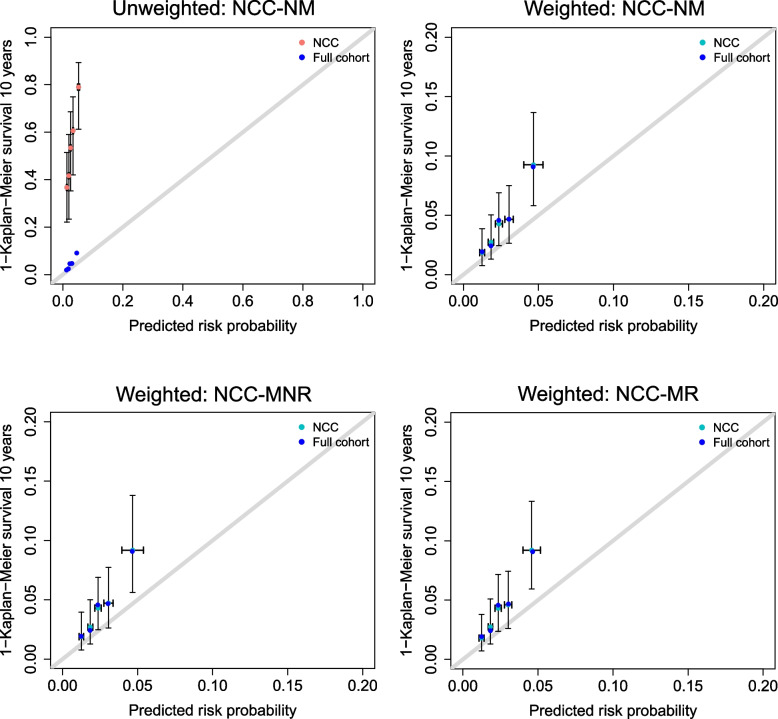
Calibration plots for the BOADICEA model applied to NCC datasets. The full cohort and the NCC datasets were divided into 5 quantiles based on predicted risk probabilities. Event estimates of the full cohort are depicted in dark blue. Unweighted event estimates are depicted in salmon; weighted event estimates are depicted in light blue. Reported 95% confidence intervals were computed by considering the variance of the Kaplan–Meier estimates, and of the mean risk probability of each group within each NCC dataset, together with the variance of these estimates between all 100 samples of NCC datasets. NCC-NM: a regular NCC design with incidence density sampling and without any matching variables; NCC-MNR: an NCC design with incidence density sampling and matching on an administrative variable, which is not associated with the model predictions; NCC-MR: NCC design with incidence density sampling and matching based on the non-genetic risk predictions

**Fig. 6:**
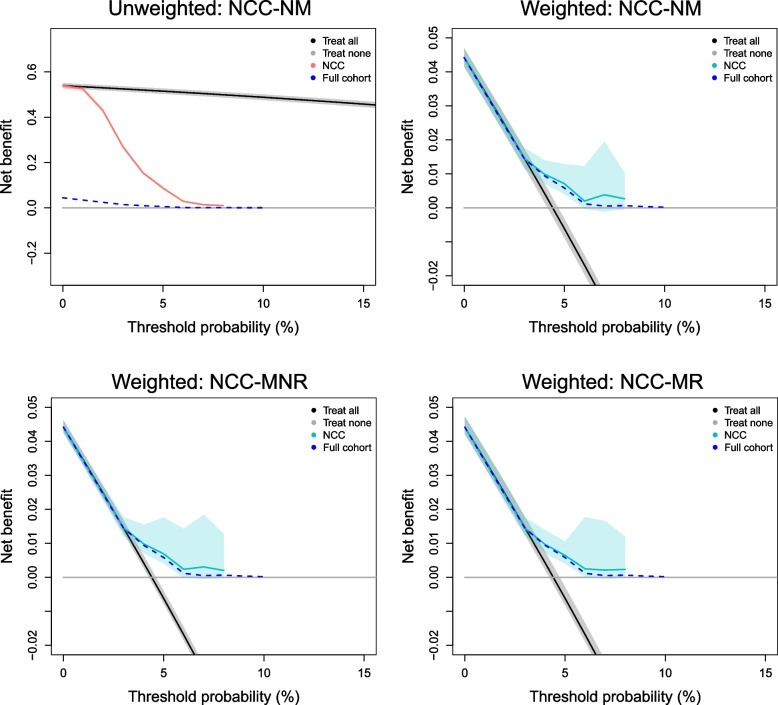
Decision curves obtained for the BOADICEA model in the full and the NCC datasets. Unweighted net benefit in the NCC datasets is depicted in salmon; weighted net benefit is depicted in light blue. These net benefit estimates correspond to the average of the estimates obtained in the 100 samples of NCC datasets. Shaded areas correspond to the bootstrap-percentile 95% confidence interval obtained across all 100 samples of NCC datasets. The net benefit of the full cohort is depicted by the dashed dark blue line. The net benefit of screening everyone is depicted in black (“Treat all”), and the net benefit of screening no one is depicted in gray (“Treat none”). The net benefit of “Treat all” is unweighted in the unweighted plot and weighted in the remaining plots. NCC-NM: a regular NCC design with incidence density sampling and without any matching variables; NCC-MNR: an NCC design with incidence density sampling and matching on an administrative variable, which is not associated with the model predictions; NCC-MR: NCC design with incidence density sampling and matching based on the non-genetic risk predictions

We evaluated the performance of the model in 3 scenarios with variations in the NCC sampling design; all sampling designs led to NCC datasets with 163 cases and 163 controls. To evaluate model predictions, we first analyzed performance metrics without setting any risk threshold. Fig. 4 clearly shows that the unweighted performance metrics obtained in the NCC datasets do not correspond to those obtained in the full cohort. The bias was larger when controls were matched to cases on a variable that is associated with the model predictions, but precision was similar for most metrics. In contrast, weighted performance metrics were unbiased in all scenarios. The results were very similar for other types of sampling weights (GLM and GAM weights, Supplementary Fig. 3). The unweighted calibration plot suggests that the model substantially underestimated the risk of the outcome (Fig. 5), while all weighted calibration plots resemble the calibration plot of the full cohort. Similarly, the unweighted decision curve indicates that the model is not more useful than the screen-all strategy, while the weighted decision curves are very similar to the decision curve on the full cohort (Fig. 6); therefore correctly showing that the model outperforms the screen-all strategy. Precision for lower probability ranges was slightly higher for the matched NCC datasets. However, for all designs the width of the confidence intervals and bias of net benefit estimates substantially increased with increasing risk thresholds, because there were fewer subjects with higher risk predictions, and therefore more variability across different realizations of the NCC sampling.

**Fig. 7:**
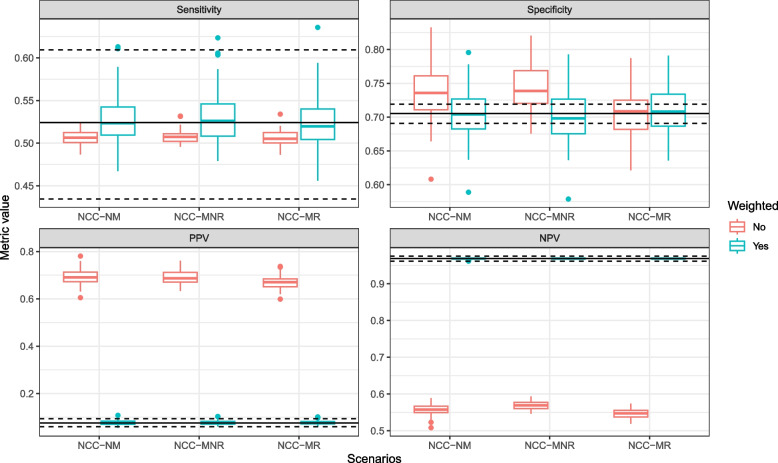
Threshold-based performance metrics obtained in the full cohort and in the NCC datasets. Here, the BOADICEA model was applied to the subjects, and those with a risk prediction lower than 3% were classified as low-risk. The horizontal black line indicates the value of the performance metric in the full cohort and the dashed horizontal black lines indicate the upper and lower bounds of the 95% confidence interval of the performance metric in the full cohort. The color of the boxplots indicates whether performance metrics of the NCC datasets are weighted (“Yes”) or unweighted (“No”). PPV: Positive predictive value, NPV: Negative predictive value, NCC-NM: a regular NCC design with incidence density sampling and without any matching variables; NCC-MNR: an NCC design with incidence density sampling and matching on an administrative variable, which is not associated with the model predictions; NCC-MR: NCC design with incidence density sampling and matching based on the non-genetic risk predictions

As with the previous performance metrics, the threshold-dependent metrics (SE, SP, PPV and NPV) were biased without weighting, and close to unbiased when weighted (Fig. 7). Unbiased estimates were also obtained for all weighted metrics when 2 controls were sampled per case; however, the precision of performance estimates was higher for most performance metrics (Supplementary Table 3, Supplementary Figs. 4 and 5). Lastly, model comparisons were also biased when unweighted metrics were used (Supplementary Fig. 6). Namely, using unweighted metrics to compare the BOADICEA model with the genetic component *or* without leads to a substantial underestimation of the benefit of the genetic component: the difference between the unweighted C-indexes of the two models was respectively 7% and 4% in the NCC-NM and NCC-MR scenarios, compared to 10% when the difference in C-index was computed using weighted metrics in either scenario, or based on the full cohort.

## Discussion

We systematically proposed how to validate prediction models in nested case–control data, by adjusting discriminative and calibration metrics, as well as calibration plots and decision curves. Despite their sample size being much smaller, we showed that NCC datasets can be used to obtain performance estimates that correspond to those of the original population-based cohorts, as long as performance metrics are appropriately weighted. The weighting procedure consists of estimating the sampling weight [20] of each subject in the NCC dataset and using those weights when computing each performance metric.

We illustrated the importance and validity of the described weighted metrics in a real-world case study where we validated the BOADICEA model in NCC datasets derived from the Rotterdam study. Although the BOADICEA model had previously been externally validated in the Rotterdam study [18], we showed that, if the model input variables had not been available for the entire cohort, unbiased performance estimates could have also been obtained in an NCC subset of the original cohort: the median/mean of the estimated metrics in the NCC datasets corresponded to their values in the Rotterdam study (Figs. 4–7).

NCC datasets are therefore a more cost-effective design for model validation, as they allow the estimation of unbiased performance metrics with a much smaller sample size: in this case less than 10% of the original cohort. This is of particular interest for evaluating the performance of models that require measuring biomarkers that are difficult to obtain in large cohorts, such as the polygenic risk scores included in the BOADICEA model. Of note, the smaller sample size of NCC datasets leads to higher uncertainty regarding the estimated performance metrics. If this is of concern, the number of controls sampled per case can be increased to increase precision of the estimates (Supplementary Table 3, Supplementary Figs. 4 and 5).

We observed two distinct patterns depending on whether metrics directly depend on the outcome prevalence or not. Metrics such as the O/E ratio, net benefit, PPV and NPV are easily flagged as implausible if unweighted. For example, the unweighted calibration plot suggests that the model substantially underestimated the risk of the outcome (Fig. 5). On the other hand, metrics such as the C-index, calibration slope, sensitivity and specificity have plausible ranges even if unweighted.

However, we illustrated that using such metrics without weighting would lead to incorrect conclusions regarding model performance. In particular, we showed that when no weighting was applied, the C-index of the BOADICEA model was consistently underestimated in the NCC datasets. This underestimation has been observed in other studies^34,38^, and is, in fact, expected, as the NCC matching procedure (on time and on potential additional matching variables) frequently attenuates the association between the outcome and any model variables that might be associated with time/matching variables. This in turn decreases discriminative performance metrics. Conclusions regarding model calibration based on unweighted metrics would also be misleading: the unweighted calibration slope was lower than 1 in all scenarios, suggesting that the model was overfitted (Fig. 4), while the model was, in reality, underfitted. Furthermore, comparisons of the predictive ability of the different components of the model (non-genetic risk estimates, genetic risk estimates and combined model) would also lead to different conclusions if metrics are not weighted (Supplementary Fig. 6). Namely, in the NCC datasets matched based on risk factors, the improvement in predictive ability associated with adding the genetic component to the model was 4% based on unweighted C-indexes, compared to 10% based on the weighted or full cohort C-indexes. This observation is important as the NCC study design is frequently used to study the added value of biomarkers that are too expensive to collect in the full cohort. Our real-world example shows that it is essential to use weighted metrics to correctly estimate the improvement of performance metrics associated with novel biomarkers.

We have also shown that unbiased performance metrics can be obtained across different NCC sampling designs (with and without matching). This indicates that the effect of different types of biases introduced during cohort sampling can be mitigated during model validation by adjusting the performance metrics with sampling weights that account for the study design. Of note, while ignoring matching on administrative variables in the weight computation has a smaller impact than ignoring matching on variables associated with model risk predictions, matching on administrative variables should still be accounted for, if possible. Other studies that have investigated weighted metrics such as the C-index and calibration slope in matched NCC designs have shown biased estimation under fine matching [4, 35]. We have not observed this in our study but the matching we employed was not as fine: there were only 3 categories when we matched based on entry into the Rotterdam study (NCC-MNR), and 6 categories when based on non-genetic risk estimates (NCC-MR), with subjects being well distributed among these categories. Moreover, Ganna et al. [4] evaluated a conditional logistic regression model that included the matching variables as predictors; meaning that they could not be included in the linear predictors used for risk estimation. This contributed to additional bias in the performance metrics obtained in the matched NCC design in their study. This was not the case with the BOADICEA model.

Finally, we have shown that unbiased performance metrics can be obtained using different methods for calculating the sampling weights (Supplementary Fig. 3), which means that weighted metrics are robust with respect to the computation method used for the weights. Similar conclusions regarding weight computation methods had already been demonstrated for the development of prediction models in NCC datasets [10, 20], but not for their validation.

This study has some limitations. We illustrated the importance of using weighted performance metrics in a large real-world cohort, where using a nested case–control design can substantially reduce the cost of validating a prediction model that requires expensive data collection. However, the use of a real-world dataset prevents us from studying the robustness of our conclusions to variations in parameters such as outcome incidence and the magnitude of the performance metrics of the model. A simulation study [35], which investigated this for a subset of the performance metrics, showed that the C-index was consistent for different outcome incidence rates, but calibration metrics deteriorated for lower incidence rates (5%). This slightly contrasts with our study, where the outcome cumulative incidence was 4.4% (95% CI 3.7–5.1%) and all metrics could be unbiasedly estimated. However, we focused on external validation of the model while Lee et al. [35] reported fivefold cross-validation metrics for the calibration metrics which might partially explain their observed decline in performance for lower outcome incidence.

We have extensively covered the most commonly recommended metrics to assess the discrimination, calibration and clinical utility of prediction models [23]; however, there are other metrics and extensions that could be used. For example, goodness-of-fit tests such as Grønnesby and Borgan or Hosmer and Lemeshow’s test are employed to assess model calibration, although due to their limitations they are falling into disuse. The use of such tests in NCC datasets has been described in the literature [4, 8]. Quantification of the incremental value of additional predictors can be estimated with the net reclassification index, and a weighted version is provided in [4]. However, concerns have also been raised regarding this metric [36].

Furthermore, for simplicity, we did not account for competing risks in our real-world illustration, as this would require using performance metrics that are adjusted to the competing risk scenario [37], and modifying the sampling procedure of the NCC study to account for competing risks [38]. In fact, with strong competing risks or presence of multiple outcomes, the case-cohort design is preferred to the NCC design. Weighting of performance metrics in this sampling design is also needed, although recommended methods to compute sampling weights in this design [39] are different than the ones described in our study.

Despite the above limitations, the described weighted metrics should be relevant for a wide audience (e.g., clinicians, machine learning practitioners, epidemiologists and biostatisticians), as they must be used for a correct performance evaluation of any type of model that estimates risk probabilities in an NCC study, from the widely used Cox model to machine learning or deep learning methods. Furthermore, difficulties in model performance evaluation, which resemble the ones we discussed, are addressed in the literature under different names (such as covariate shift in the machine learning field [40]). The NCC sampling design is a special case within these concepts, as it performs sampling in a cost-effective way.

In summary, this study provides clear guidance on how prediction models should be validated in NCC studies using relevant performance metrics. Previously, most of this information was scattered in the literature and not available for all the metrics. Namely, weighted versions of performance metrics are available in different R packages [8], but we had to implement weight adjustments for threshold-based metrics and decision curve analyses for survival data. The code to implement the adjustment of these performance metrics with sampling weights is now available in a GitHub repository (see section “code availability”) for easy implementation and should facilitate the adoption of weighted metrics also by non-specialists.

## Conclusions

Clinical prediction models can be validated in NCC studies if the performance metrics are appropriately adjusted using the sampling weights of the subjects in the NCC dataset. If performance metrics are not weighted, their estimates will be biased, with the magnitude of the bias being higher when matching variables are correlated with the model predictions. The choice of the method to compute sampling weights does not lead to large changes in the estimated weighted metrics, as long as the sampling design and all matching variables are considered in the computation. These results are particularly relevant for the validation of models that predict rare outcomes, and whose input variables cannot be measured in all subjects in the validation cohort.

## Supplementary Information

**Supplementary Material 1.**

## Data Availability

The dataset analyzed in this study is not publicly available due to privacy regulations; however data can be obtained upon request to the management team of the Rotterdam Study (datamanagement.ergo@erasmusmc.nl).

## Abbreviations

BOADICEA: Breast and Ovarian Analysis of Disease Incidence and Carrier Estimation Algorithm
CI: Confidence Interval
GAM: Generalized additive model
GLM: Generalized linear model
KM: Kaplan–Meier
NB: Net benefit
NCC: Nested case–control
NCC-MNR: An NCC design with incidence density sampling and matching on an administrative variable
NCC-MR: An NCC design with incidence density sampling and matching based on the non-genetic risk estimates
NCC-NM: Regular NCC design with incidence density sampling and without any matching variables
NPV: Negative predictive value
O/E ratio: Observed-to-expected events ratio
PPV: Positive predictive value
PRS: Polygenic risk score
RF: Risk factors
SE: Sensitivity
SP: Specificity

## References

[1] McCarthy CE, Bonnet LJ, Marcus MW, Field JK. Development and validation of a multivariable risk prediction model for head and neck cancer using the UK Biobank. Int J Oncol. 2020;57(5):1192–202. 33491742 10.3892/ijo.2020.5123

[2] Cederholm J, Eeg-Olofsson K, Eliasson B, Zethelius B, Nilsson PM, Gudbjörnsdottir S. Risk prediction of cardiovascular disease in type 2 diabetes: a risk equation from the Swedish National Diabetes Register. Diabetes Care. 2008;31(10):2038–43. 18591403 10.2337/dc08-0662 PMC2551651

[3] Moons KGM, Kengne AP, Woodward M, Royston P, Vergouwe Y, Altman DG, et al. Risk prediction models: I. Development, internal validation, and assessing the incremental value of a new (bio)marker. Heart. 2012;98(9):683–90. 22397945 10.1136/heartjnl-2011-301246

[4] Ganna A, Reilly M, De Faire U, Pedersen N, Magnusson P, Ingelsson E. Risk prediction measures for case-cohort and nested case-control designs: An application to cardiovascular disease. Am J Epidemiol. 2012;175(7):715–24. 22396388 10.1093/aje/kwr374 PMC3324433

[5] Salim A, Delcoigne B, Villaflores K, Koh WP, Yuan JM, van Dam RM, et al. Comparisons of risk prediction methods using nested case-control data. Stat Med. 2017;36(3):455–65. 27734520 10.1002/sim.7143

[6] Biesheuvel CJ, Vergouwe Y, Oudega R, Hoes AW, Grobbee DE, Moons KGM. Advantages of the nested case-control design in diagnostic research. BMC Med Res Methodol. 2008;8(1):1–7. 18644127 10.1186/1471-2288-8-48 PMC2500041

[7] Moons KGM, Van Klei W, Kalkman CJ. Preoperative risk factors of intraoperative hypothermia in major surgery under general anesthesia. Anesth Analg. 2003;96(6):1843–4. 12761031 10.1213/01.ANE.0000063178.15467.D8

[8] Choudhury PP, Maas P, Wilcox A, Wheeler W, Brook M, Check D, et al. iCARE: An R package to build, validate and apply absolute risk models. PLoS ONE. 2020;15(2):e0228198. 32023287 10.1371/journal.pone.0228198 PMC7001949

[9] Kim RS. Analysis of Nested Case-Control Study Designs: Revisiting the Inverse Probability Weighting Method. Communications for Statistical Applications and Methods. 2013;20(6):455–66. 28503512 10.5351/CSAM.2013.20.6.455 PMC5426119

[10] Borgan Ø, Keogh R. Nested case–control studies: should one break the matching? Lifetime Data Anal. 2015;21(4):517–41. 25608704 10.1007/s10985-015-9319-y

[11] Zelic R, Zugna D, Bottai M, Andrén O, Fridfeldt J, Carlsson J, et al. Estimation of Relative and Absolute Risks in a Competing-Risks Setting Using a Nested Case-Control Study Design: Example from the ProMort Study. Am J Epidemiol. 2019;188(6):1165–73. 30976789 10.1093/aje/kwz026 PMC8210820

[12] Delcoigne B, Colzani E, Prochazka M, Gagliardi G, Hall P, Abrahamowicz M, et al. Breaking the matching in nested case–control data offered several advantages for risk estimation. J Clin Epidemiol. 2017;1(82):79–86. 10.1016/j.jclinepi.2016.11.014 27923734

[13] Rentroia-Pacheco B, Tokez S, Bramer EM, Venables ZC, Van De Werken HJG, Bellomo D, et al. Personalised decision making to predict absolute metastatic risk in cutaneous squamous cell carcinoma: development and validation of a clinico-pathological model. eClinicalMedicine. 2023;63:102150. 37662519 10.1016/j.eclinm.2023.102150 PMC10468358

[14] Murphy JD, Olshan AF, Lin FC, Troester MA, Nichols HB, Butt J, et al. A Predictive Model of Noncardia Gastric Adenocarcinoma Risk Using Antibody Response to Helicobacter pylori Proteins and Pepsinogen. Cancer Epidemiol Biomark Prev. 2022;31(4):811–20. 10.1158/1055-9965.EPI-21-0869 PMC8983566 35131882

[15] Hoogeveen RM, Pereira JPB, Nurmohamed NS, Zampoleri V, Bom MJ, Baragetti A, et al. Improved cardiovascular risk prediction using targeted plasma proteomics in primary prevention. Eur Heart J. 2020;41(41):3998–4007. 32808014 10.1093/eurheartj/ehaa648 PMC7672529

[16] Reps JM, Ryan PB, Rijnbeek PR, Schuemie MJ. Design matters in patient-level prediction: evaluation of a cohort vs. case-control design when developing predictive models in observational healthcare datasets. Journal of Big Data. 2021;8(1):1–18.33425651

[17] Lee A, Mavaddat N, Wilcox AN, Cunningham AP, Carver T, Hartley S, et al. BOADICEA: a comprehensive breast cancer risk prediction model incorporating genetic and nongenetic risk factors. Genet Med. 2019;21(8):1708–18. 30643217 10.1038/s41436-018-0406-9 PMC6687499

[18] Lakeman IMM, Rodríguez-Girondo M, Lee A, Ruiter R, Stricker BH, Wijnant SRA, et al. Validation of the BOADICEA model and a 313-variant polygenic risk score for breast cancer risk prediction in a Dutch prospective cohort. Genet Med. 2020;22(11):1803–11. 32624571 10.1038/s41436-020-0884-4 PMC7605432

[19] Zhou QM, Wang X, Zheng Y, Cai T. New weighting methods when cases are only a subset of events in a nested case-control study. Biom J. 2022;64(7):1240–59. 35754309 10.1002/bimj.202100194 PMC10249867

[20] Støer NC, Samuelsen SO. MultipleNCC: Inverse probability weighting of nested case-control data. R Journal. 2016;8(2):5–18.

[21] Langholz B, Richardson D. Are Nested Case-Control Studies Biased? Epidemiology. 2009;20(3):321–9. 19289963 10.1097/EDE.0b013e31819e370b PMC4194079

[22] Støer NC, Samuelsen SO. Comparison of estimators in nested case-control studies with multiple outcomes. Lifetime Data Anal. 2012;18(3):261–83. 22382602 10.1007/s10985-012-9214-8

[23] Moons KGM, Altman DG, Reitsma JB, Ioannidis JPA, Macaskill P, Steyerberg EW, et al. Transparent reporting of a multivariable prediction model for individual prognosis or diagnosis (TRIPOD): Explanation and elaboration. Ann Intern Med. 2015;162(1):W1–73. 25560730 10.7326/M14-0698

[24] Steyerberg EW. Clinical Prediction Models: A Practical Approach to Development, Validation, and Updating Second Edition. New York: NY: Springer Nature Switzerland; 2019. (Statistics for Biology and Health).

[25] Vickers AJ, Elkin EB. Decision curve analysis: A novel method for evaluating prediction models. Med Decis Making. 2006;26(6):565–74. 17099194 10.1177/0272989X06295361 PMC2577036

[26] Parikh R, Mathai A, Parikh S, Sekhar GC, Thomas R. Understanding and using sensitivity, specificity and predictive values. Indian J Ophthalmol. 2008;56(1):45–50. 18158403 10.4103/0301-4738.37595 PMC2636062

[27] Van Calster B, Nieboer D, Vergouwe Y, De Cock B, Pencina MJ, Steyerberg EW. A calibration hierarchy for risk models was defined: From utopia to empirical data. J Clin Epidemiol. 2016;1(74):167–76. 10.1016/j.jclinepi.2015.12.005 26772608

[28] McLernon DJ, Giardiello D, Van Calster B, Wynants L, van Geloven N, van Smeden M, et al. Assessing Performance and Clinical Usefulness in Prediction Models With Survival Outcomes: Practical Guidance for Cox Proportional Hazards Models. Ann Intern Med. 2023;176(1):105–14. 36571841 10.7326/M22-0844

[29] Vickers AJ, Cronin AM, Elkin EB, Gonen M. Extensions to decision curve analysis, a novel method for evaluating diagnostic tests, prediction models and molecular markers. BMC Medical Inform Decis Mak. 2008;8:53. 10.1186/1472-6947-8-53 PMC2611975 19036144

[30] Pfeiffer RM, Gail MH. Estimating the decision curve and its precision from three study designs. Biom J. 2020;62(3):764–76. 31394013 10.1002/bimj.201800240 PMC8620346

[31] Wieczorek J, Guerin C, McMahon T. K-fold cross-validation for complex sample surveys. In: In: Stat. John Wiley & Sons, Ltd; 2022. p. e454.

[32] Arnold M, Morgan E, Rumgay H, Mafra A, Singh D, Laversanne M, et al. Current and future burden of breast cancer: Global statistics for 2020 and 2040. Breast. 2022;1(66):15–23. 10.1016/j.breast.2022.08.010 PMC9465273 36084384

[33] Marmot M, Altman DG, Cameron DA, Dewar JA, Thompson SG, Wilcox M. The benefits and harms of breast cancer screening: an independent review. The Lancet. 2012;380(9855):1778–86. 10.1016/S0140-6736(12)61611-0 23117178

[34] Ikram MA, Brusselle G, Ghanbari M, Goedegebure A, Ikram MK, Kavousi M, et al. Objectives, design and main findings until 2020 from the Rotterdam Study. European J Epidemiol. 2020;35(5):483–517. 32367290 10.1007/s10654-020-00640-5 PMC7250962

[35] Lee M, Zeleniuch-Jacquotte A, Liu M. Empirical evaluation of sub-cohort sampling designs for risk prediction modeling. J Appl Stat. 2021;48(8):1374–401. 35706464 10.1080/02664763.2020.1861225 PMC9042011

[36] Pepe MS, Fan J, Feng Z, Gerds T, Hilden J. The Net Reclassification Index (NRI): A Misleading Measure of Prediction Improvement Even with Independent Test Data Sets. Stat Biosci. 2015;7(2):282–95. 26504496 10.1007/s12561-014-9118-0 PMC4615606

[37] Ramspek CL, Teece L, Snell KIE, Evans M, Riley RD, Van Smeden M, et al. Lessons learnt when accounting for competing events in the external validation of time-To-event prognostic models. Int J Epidemiol. 2022;51(2):615–25. 34919691 10.1093/ije/dyab256 PMC9082803

[38] Wolkewitz M, Cooper BS, Palomar-Martinez M, Olaechea-Astigarraga P, Alvarez-Lerma F, Schumacher M. Nested case-control studies in cohorts with competing events. Epidemiology. 2014;25(1):122–5. 24240653 10.1097/EDE.0000000000000029

[39] Sanderson J, Thompson SG, White IR, Aspelund T, Pennells L. Derivation and assessment of risk prediction models using case-cohort data. BMC Med Res Methodol. 2013;13:113. 24034146 10.1186/1471-2288-13-113 PMC3848813

[40] Sugiyama M, Krauledat M, Müller KR. Covariate shift adaptation by importance weighted cross validation. J Mach Learn Res. 2007;8:985–1005.

