# Supplementary material for: Weighted metrics are required when evaluating the performance of prediction models in nested case–control studies
Source: BMC Med Res Methodol. 2024 May 17;24:115. doi: 10.1186/s12874-024-02213-6 (PMC11533296; doi:10.1186/s12874-024-02213-6)
Supplement: Supplementary file 1 — Supplementary Material 1. [file 12874_2024_2213_MOESM1_ESM.docx]

# Supplementary Material

**Supplementary Table 1** **R functions and respective packages for validating prediction models in NCC cohorts**. Threshold-based metrics include sensitivity, specificity, positive predictive value and negative predictive value. Our custom functions are available in a GitHub repository.

| **Data type** | **Analyses** | **Full cohort** | | **NCC adaptation** | |
| --- | --- | --- | --- | --- | --- |
|  |  | **function(s)** | **package** | **function(s)** | **package** |
|  | Computing sampling weights | *-* | *-* | *KMprob*  *GAMprob*  *GLMprob* | *multipleNCC*  (20) |
| **Binary data** | Area under the ROC curve | *coords* | *pROC*  (40) | *WeightedROC,* with sampling weights provided in weight argument, and *WeightedAUC* | *WeightedROC*  (41) |
|  | Threshold-based metrics | *coords* | *pROC*  (40) | *sens,spec,ppv and npv functions,* with sampling weights provided in *case_weights* argument | *yardstick*  (42) |
|  | Mean calibration & calibration slope | *glm*, family = binomial | stats | *glm*, family = binomial, with sampling weights provided in *weights* argument | *stats* |
|  | Decision curve | *dca* | *Dcurves*  (43) | *dca* with prevalence parameter | *dcurves*  (43) |
| **Survival data** | C-index | *rcorr.cens* | *Hmisc*  (44) | *cIndex* , with sampling weights provided in *weight* argument | *intsurv*  (45) |
|  | Threshold-based metrics | *survivalROC* | *survivalROC*  (46) | custom function | |
|  | Mean calibration | *survfit* for observed events*,* mean of predicted risk | *Survival*  (47) | *survfit* for observed events, weighted mean of predicted risk | *survival*  (47) |
|  | Calibration slope | *coxph* | *survival*  (47) | *coxph,* with weights parameter | *survival*  (47) |
|  | Decision curve | *dca* | *dcurves*  (43) | custom function | |

**Supplementary Table 2 Performance metrics of the BOADICEA model and sub-models in the full cohort of 4,377 women.** Performance metrics were computed for risk estimates based on 1) Age alone (Age); 2) Age and Risk Factors (Age+RF); 3) Age and Polygenic Risk Score (Age+PRS); and, finally, for the BOADICEA model which uses all of the previous components (Age+RF+PRS). 95% confidence intervals based on 1000 bootstrap repetitions were computed for each metric. O/E ratio: observed to expected ratio, PPV: Positive predictive value, NPV: Negative predictive value

| **Metric** | **Age** | **Age + RF** | **Age + PRS** | **BOADICEA model** |
| --- | --- | --- | --- | --- |
| **C-index** | 0.46 (0.42-0.51) | 0.55 (0.50-0.59) | 0.63 (0.59-0.68) | 0.65 (0.61-0.69) |
| **O/E ratio** | 1.48 (1.25-1.69) | 1.76 (1.49-2.02) | 1.42 (1.20-1.62) | 1.69 (1.42-1.93) |
| **Calibration slope** | -1.22 (-2.73-0.18) | 0.69 (0.14-1.25) | 1.27 (0.87-1.72) | 1.19 (0.87-1.54) |
| **Sensitivity** | 0.33 (0.25-0.42) | 0.25 (0.18-0.33) | 0.68 (0.60-0.76) | 0.52 (0.43-0.61) |
| **Specificity** | 0.61 (0.59-0.62) | 0.80 (0.78-0.81) | 0.55 (0.53-0.56) | 0.71 (0.69-0.72) |
| **PPV** | 0.04 (0.03-0.05) | 0.05 (0.04-0.07) | 0.06 (0.05-0.08) | 0.08 (0.06-0.09) |
| **NPV** | 0.95 (0.94-0.96) | 0.96 (0.95-0.97) | 0.97 (0.97-0.98) | 0.97 (0.96-0.98) |

|  | No. Controls | Weighted C-index | Weighted O/E ratio | Weighted  Calibration  slope | Weighted  SE | Weighted  SP | Weighted  PPV | Weighted  NPV |
| --- | --- | --- | --- | --- | --- | --- | --- | --- |
| NCC-NM | 1 | 0.65  (0.62-0.69) | 1.68  (1.53-1.84) | 1.20  (0.85-1.54) | 0.53  (0.48-0.59) | 0.70  (0.64-0.78) | 0.08  (0.06-0.10) | 0.97  (0.96-0.97) |
|  | 2 | 0.65  (0.62-0.68) | 1.69  (1.60-1.79) | 1.19  (0.93-1.59) | 0.53  (0.49-0.56) | 0.70  (0.65-0.76) | 0.08  (0.07-0.09) | 0.97  (0.97-0.97) |
| NCC-MNR | 1 | 0.65  (0.61-0.68) | 1.67  (1.55-1.81) | 1.20  (0.85-1.69) | 0.53  (0.49-0.60) | 0.70  (0.65-0.77) | 0.08  (0.06-0.10) | 0.97  (0.97-0.97) |
|  | 2 | 0.65  (0.61-0.68) | 1.67  (1.59-1.76) | 1.18  (0.94-1.45) | 0.53  (0.50-0.57) | 0.70  (0.65-0.75) | 0.08  (0.07-0.09) | 0.97  (0.97-0.97) |
| NCC-MR | 1 | 0.65  (0.63-0.68) | 1.70  (1.58-1.82) | 1.25  (0.96-1.60) | 0.52  (0.47-0.58) | 0.71  (0.65-0.77) | 0.08  (0.06-0.10) | 0.97  (0.97-0.97) |
|  | 2 | 0.65  (0.63-0.68) | 1.69  (1.58-1.77) | 1.22  (0.99-1.48) | 0.52  (0.49-0.56) | 0.7  (0.66-0.76) | 0.08  (0.07-0.09) | 0.97  (0.97-0.97) |

**Supplementary Table 3** **Performance metrics obtained in the NCC cohorts, with 2 controls sampled for each case versus 1 control per case.** Sensitivity (SE), Specificity (SP), Positive predictive value (PPV) and Negative predictive value (NPV) were computed after applying the BOADICEA model to the subjects and considering those with a risk prediction lower than 3% as low-risk. O/E ratio: Observed-to-expected events ratio. NCC-NM: a regular NCC design with incidence density sampling and without any matching variables; NCC-MNR: a NCC design with incidence density sampling and matching on an administrative variable, which is not associated with the model predictions; NCC-MR: NCC design with incidence density sampling and matching based on the non-genetic risk predictions.


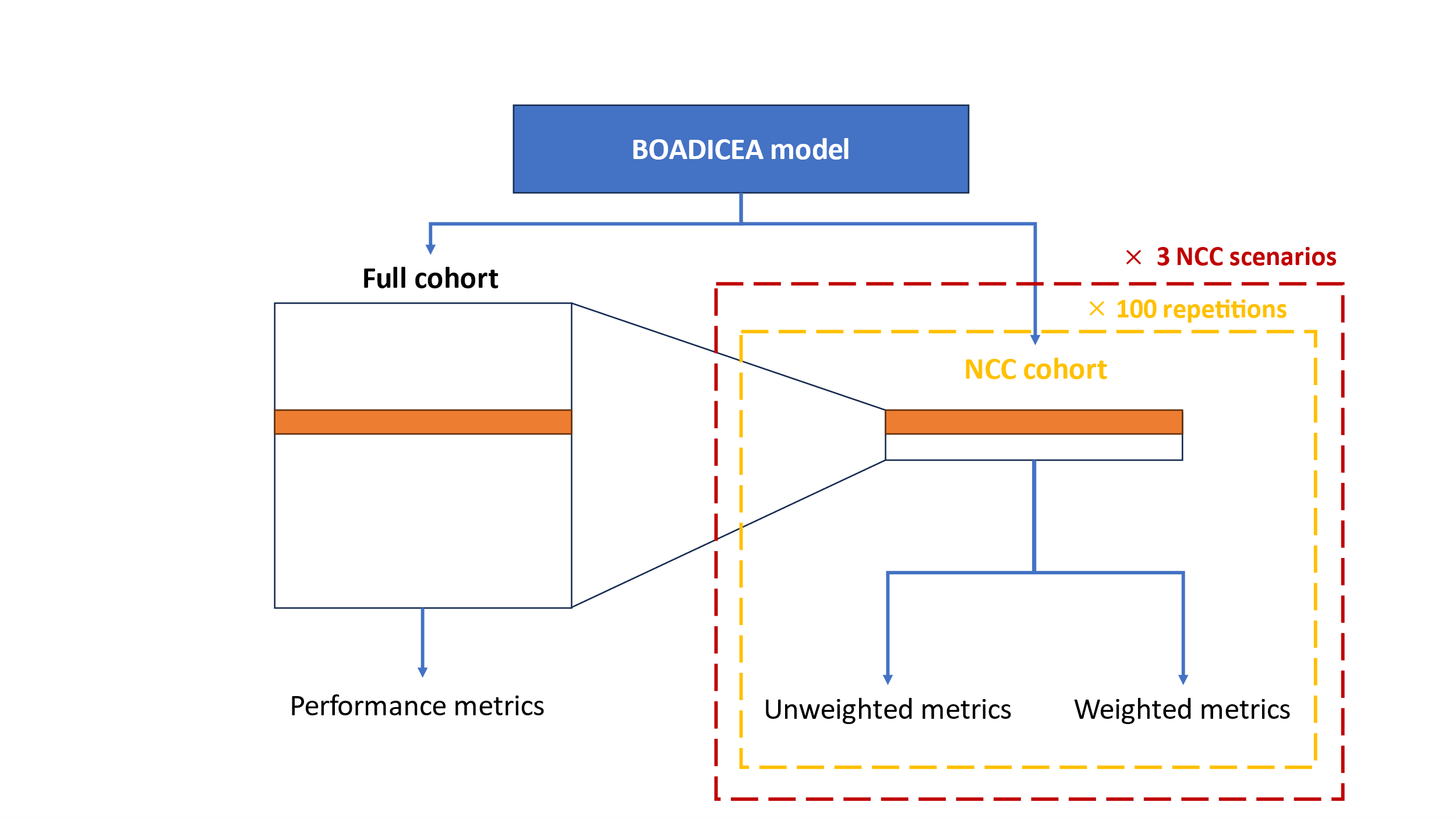


**Supplementary Figure 1** **Evaluation setup for comparison of performance metrics obtained in the full and the NCC cohorts.** Sampling of NCC cohorts from the full cohort is performed 100 times. The BOADICEA model is validated in each NCC sampling, using both unweighted and weighted performance metrics. The performance metrics obtained in the NCC cohorts are then compared with those obtained in the full cohort. The orange rectangle represents cases, and the white block represents controls.


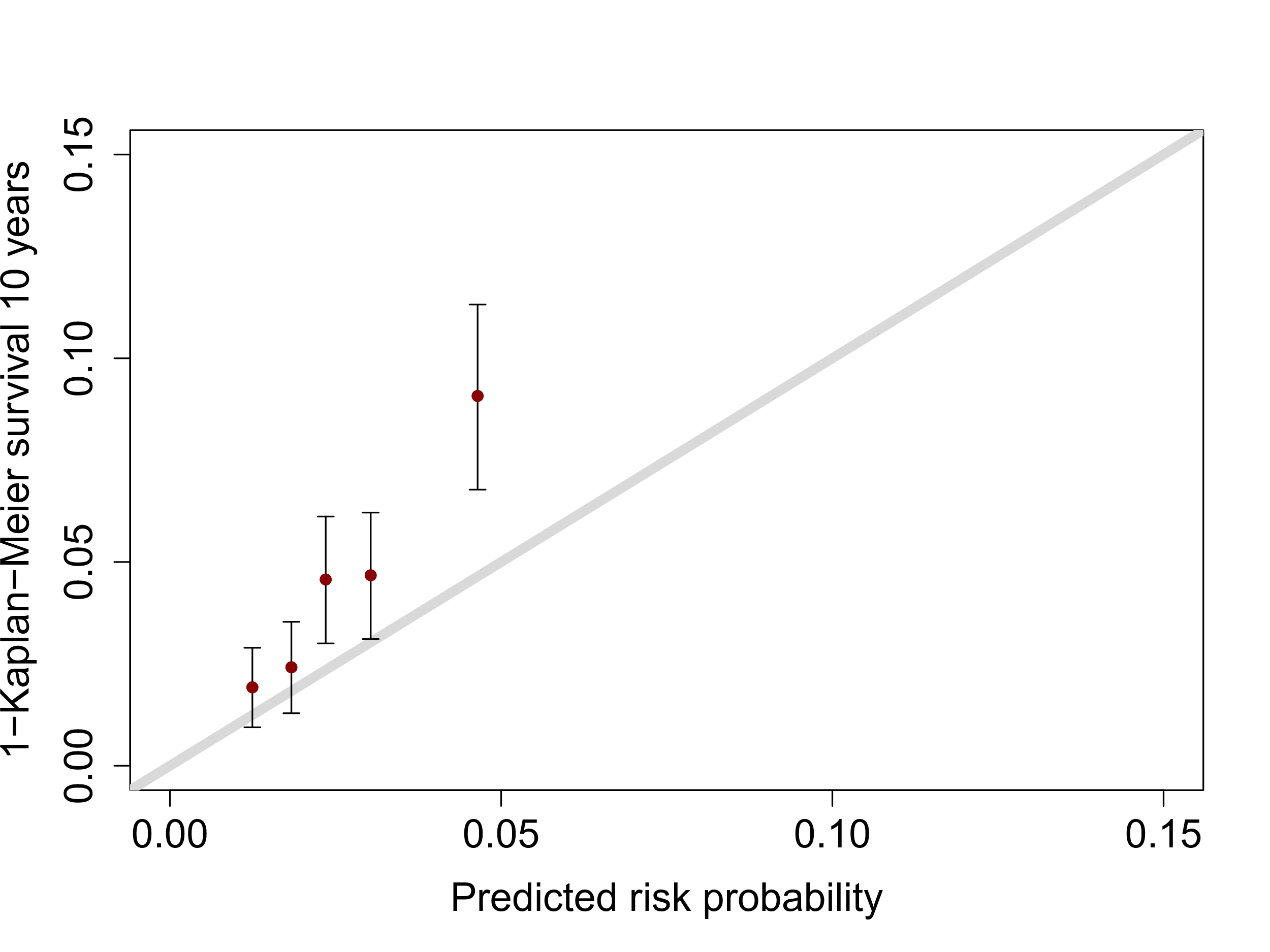


**Supplementary Figure 2 Calibration plot of the BOADICEA model applied to the Rotterdam study (full cohort)**. The full cohort was divided into 5 quantiles based on the predicted risk probabilities. Reported 95% confidence intervals correspond to the confidence intervals of the Kaplan-Meier survival estimates for each group.


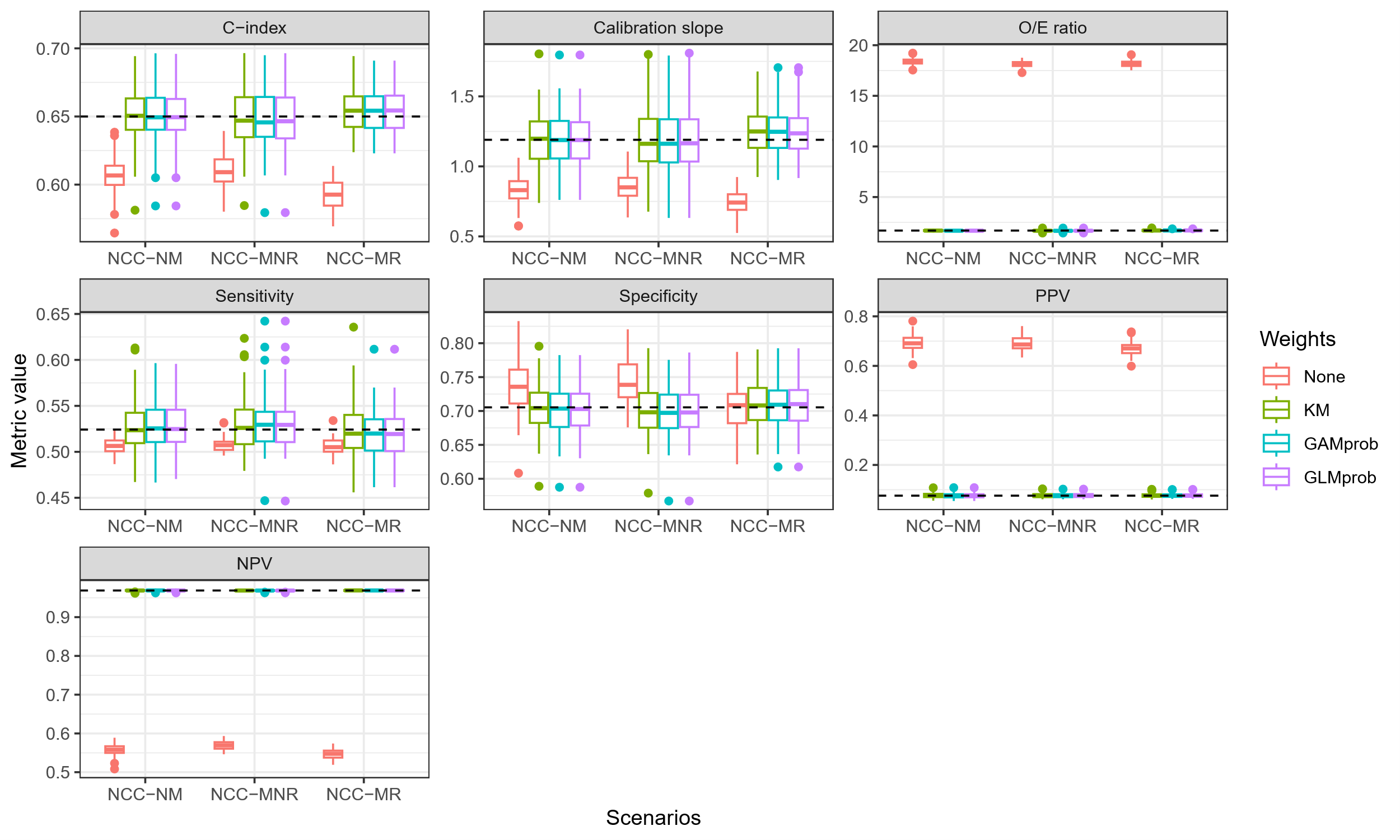


**Supplementary Figure 3** **Performance metrics obtained in the full and NCC cohorts, using different methods to calculate the sampling weights.** The dashed horizontal black line indicates the value of the performance metric in the full cohort. NCC-NM: a regular NCC design with incidence density sampling and without any matching variables; NCC-MNR: an NCC design with incidence density sampling and matching on an administrative variable, which is not associated with the model predictions; NCC-MR: NCC design with incidence density sampling and matching based on the non-genetic risk predictions. KM: Kaplan-Meier type of sampling weights. GLMprob: sampling weights obtained with logistic regression. GAMprob: sampling weights obtained with generalized additive models.


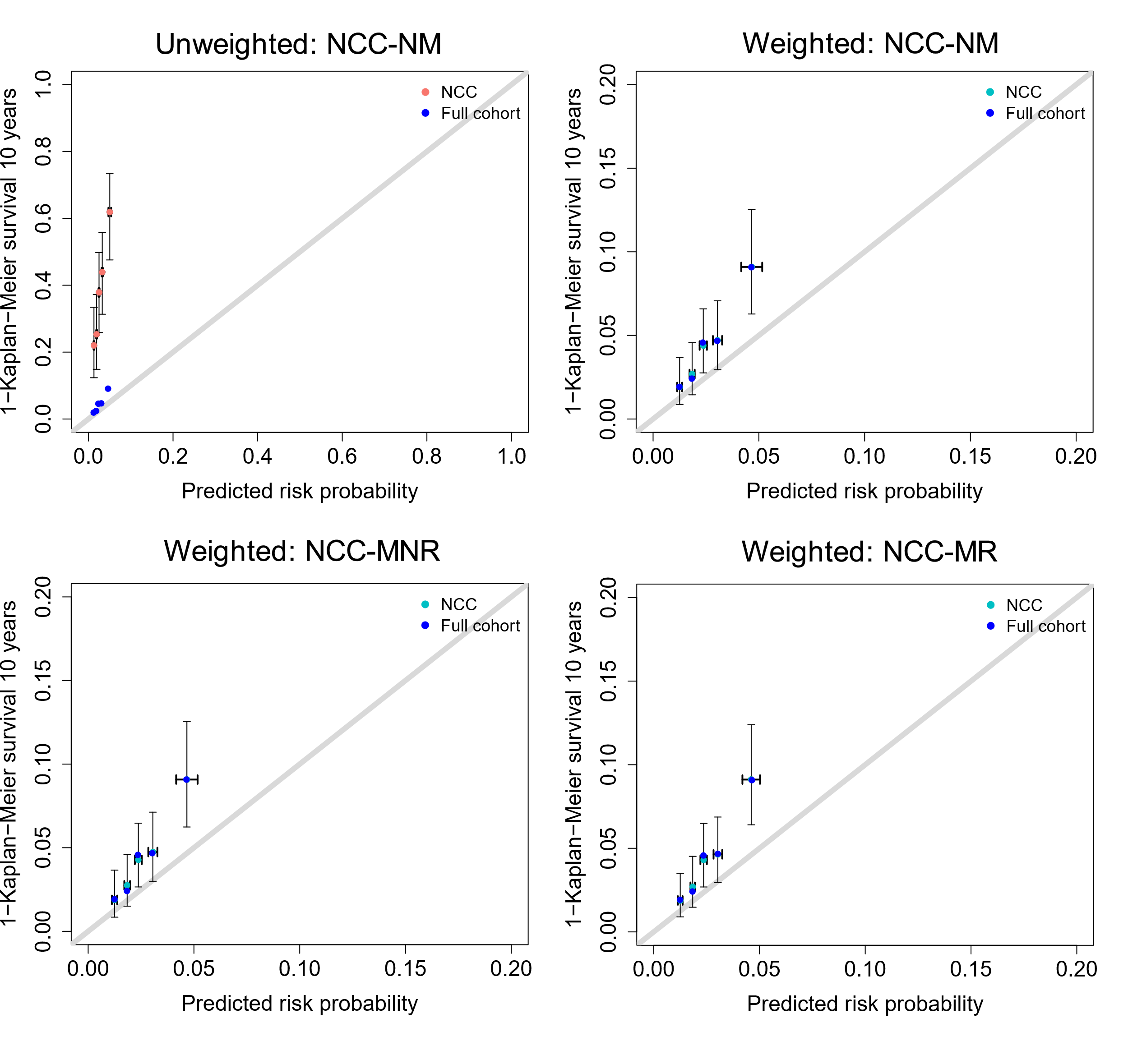


**Supplementary Figure 4 Calibration plots of the BOADICEA model applied to NCC cohorts, with 2 controls sampled per case**. The full cohort and the NCC cohorts were divided into 5 quantiles based on predicted risk probabilities. Event estimates of the full cohort are depicted in dark blue. Unweighted event estimates are depicted in salmon; weighted event estimates are depicted in light blue. Reported 95% confidence intervals were computed by considering the variance of the Kaplan-Meier estimates, and of the mean risk probability of each group, within each NCC cohort and the variance of the estimates between all 100 samples of NCC cohorts. NCC-NM: a regular NCC design with incidence density sampling and without any matching variables; NCC-MNR: an NCC design with incidence density sampling and matching on an administrative variable, which is not associated with the model predictions; NCC-MR: NCC design with incidence density sampling and matching based on the non-genetic risk predictions.


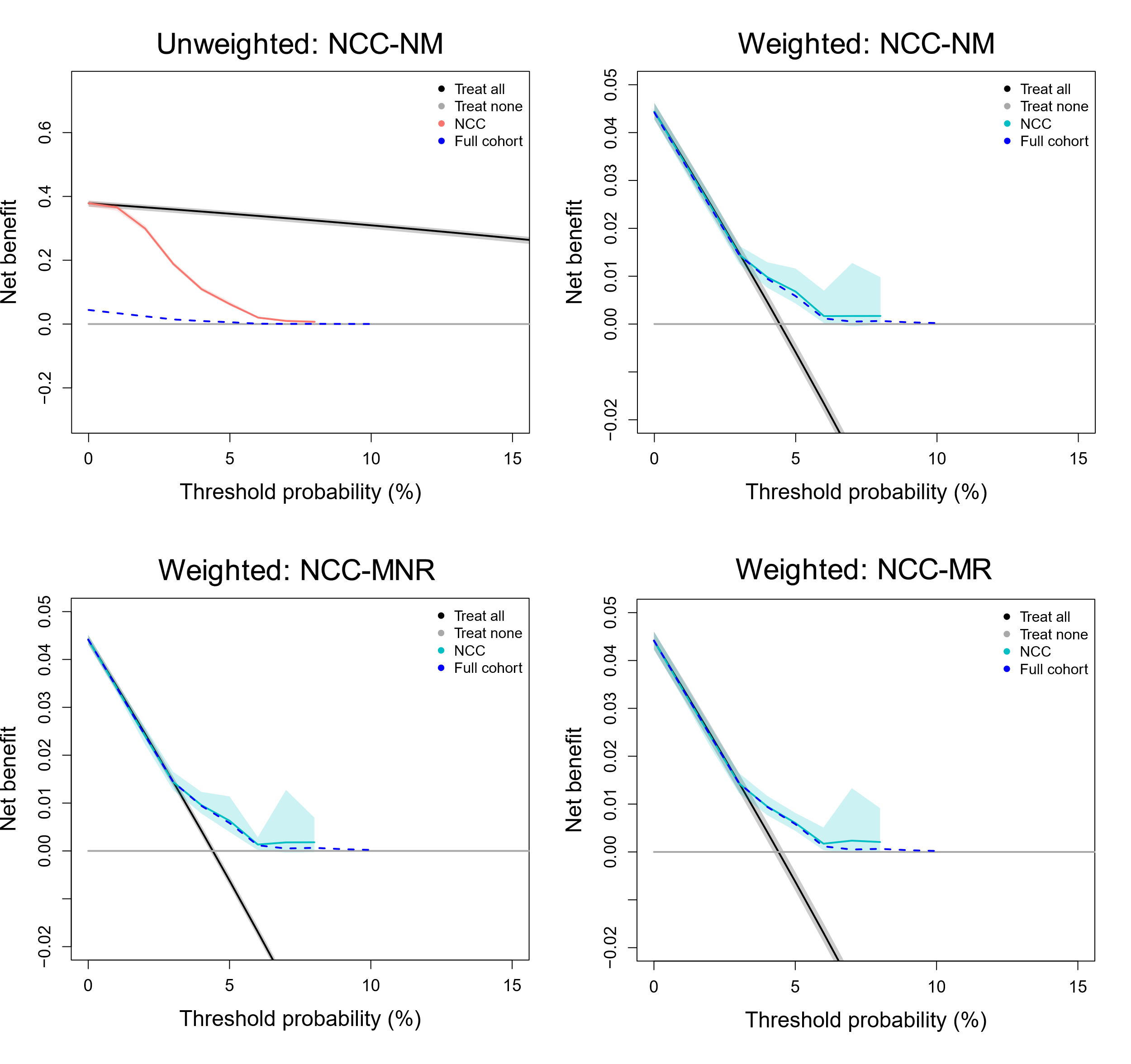


**Supplementary Figure 5 Decision curves obtained for the BOADICEA model in the full and in the NCC cohorts, with 2 controls sampled per case.** Unweighted net benefit in the NCC cohort is depicted in salmon; weighted net benefit is depicted in light blue. These net benefit estimates correspond to the average of the estimates obtained in the 100 samples of NCC cohorts. Shaded areas correspond to the bootstrap-percentile 95% confidence interval obtained across all 100 samples of NCC cohorts. The net benefit of the full cohort is depicted by the dashed dark blue line. The net benefit of screening everyone is depicted in black (“Treat all”), and the net benefit of screening no one is depicted in gray (“Treat none”). The net benefit of “Treat all” is unweighted in the unweighted plot, and weighted in the remaining plots. NCC-NM: a regular NCC design with incidence density sampling and without any matching variables; NCC-MNR: an NCC design with incidence density sampling and matching on an administrative variable, which is not associated with the model predictions; NCC-MR: NCC design with incidence density sampling and matching based on the non-genetic risk predictions.


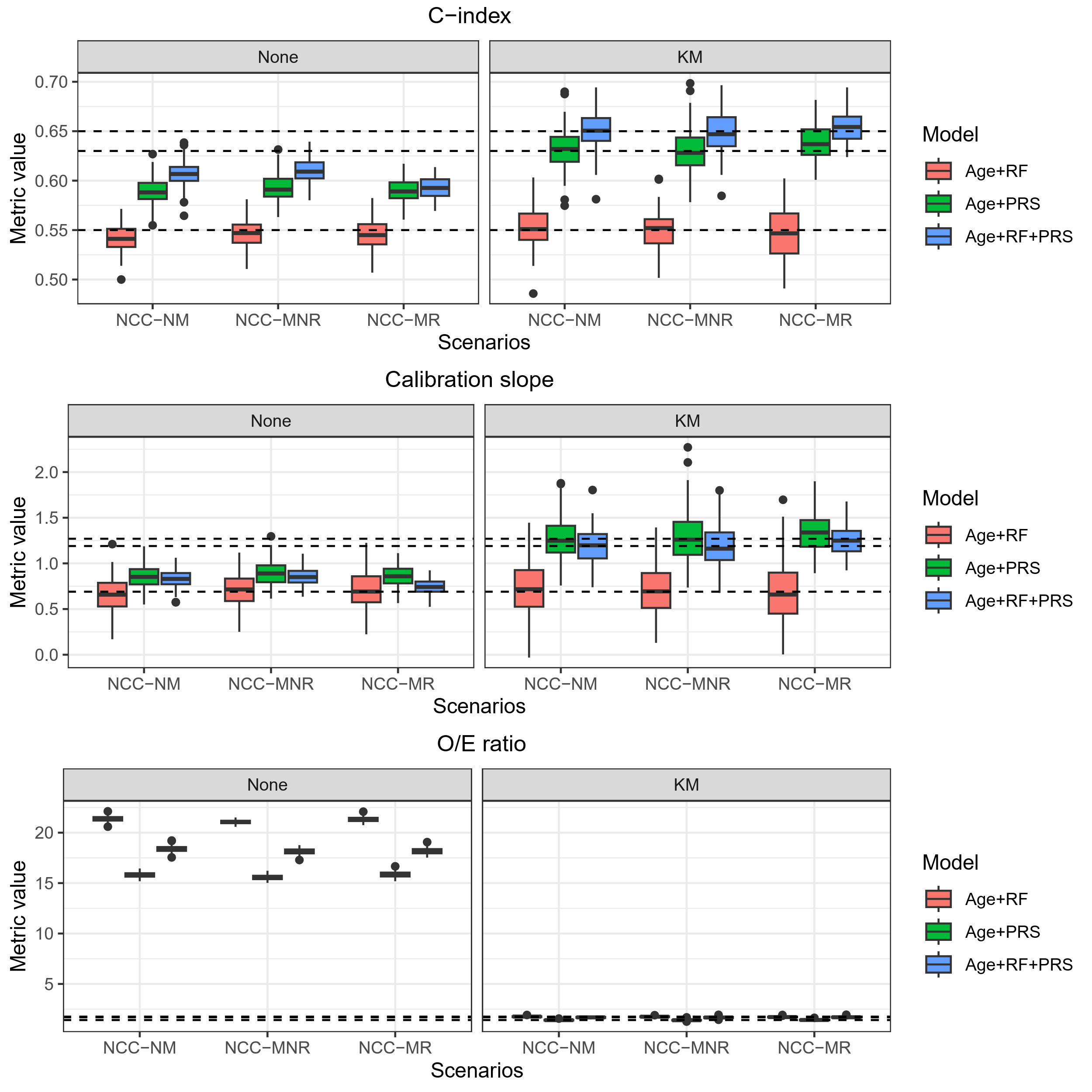


**Supplementary Figure 6 Performance metrics obtained in the full and NCC cohorts, for different BOADICEA model components.** We compare performance metrics obtained in the full cohort with those obtained with NCC cohorts, using Kaplan-Meier type sampling weights, for: 10-year risk estimates based on Age and Risk Factors (Age+RF), Age and Polygenic Risk Score (Age+PRS), and all of these components (Age+RF+PRS). NCC-NM: a regular NCC design with incidence density sampling and without any matching variables; NCC-MNR: an NCC design with incidence density sampling and matching on an administrative variable, which is not associated with the model predictions; NCC-MR: NCC design with incidence density sampling and matching based on the non-genetic risk predictions.
